# A Comprehensive Analysis of Pyroptosis-Related lncRNAs Signature Associated With Prognosis and Tumor Immune Microenvironment of Pancreatic Adenocarcinoma

**DOI:** 10.3389/fgene.2022.899496

**Published:** 2022-07-06

**Authors:** Kai Zhao, Xiangyu Li, Yuanxin Shi, Yun Lu, Peng Qiu, Zhengdong Deng, Wei Yao, Jianming Wang

**Affiliations:** ^1^ Department of Biliary and Pancreatic Surgery/Cancer Research Center Affiliated Tongji Hospital, Tongji Medical College, Huazhong University of Science and Technology, Wuhan, China; ^2^ Department of Oncology Affiliated Tongji Hospital, Tongji Medical College, Huazhong University of Science and Technology, Wuhan, China; ^3^ Affiliated Tianyou Hospital, Wuhan University of Science & Technology, Wuhan, China

**Keywords:** pancreatic adenocarcinoma, pyroptosis, lncRNAs, prognosis, tumor immune microenvironment, immunotherapy

## Abstract

**Background:** Globally, pancreatic adenocarcinoma (PAAD) is a common and highly devastating gastrointestinal malignancy that seriously threatens human health. Pyroptosis refers to an emerging form of programmed cell death that has been discovered in recent years, and studies have demonstrated that long non-coding RNA (lncRNA) may act as a moderator in the pyroptosis process of cancer cells. However, relevant explorations about lncRNAs and pyroptosis are still insufficient in PAAD. Therefore, our research is designed to make a comprehensive analysis of the potential values of pyroptosis-related lncRNAs in PAAD.

**Methods:** By integrating the RNA-sequencing, somatic mutation, and copy number variation (CNV) datasets, as well as the clinicopathological features, we established and validated a risk signature based on pyroptosis-related lncRNAs, and comprehensively analyzed its clinical significance and the potential connection with the tumor immune microenvironment (TIME).

**Consequences:** The genetic variation landscape displayed that the somatic mutations were rare while CNV changes were general and mainly concentrated on copy number amplification of these 52 pyroptosis-related genes. Subsequently, a risk signature consisting of 10 lncRNAs (TRAF3IP2-AS1, LINC00519, LINC01133, LINC02251, AC005332.6, AL590787.1, AC090114.2, TRPC7-AS1, MIR223HG, and MIR3142HG) was constructed and patients were divided into different subgroups according to the median risk score; patients with high-risk scores presented worse outcomes compared to those with low-risk scores in the training, testing, and entire cohorts. Furthermore, patients at low-risk scores possessed a higher infiltration abundance of immune cells compared with high-risk patients, which was consistent with the expression levels of lncRNAs between the high/low-risk groups. Drug sensitivity analysis showed that low-risk scores were related to anti-cancer agents like AICAR and Axitinib, whereas high-risk scores were connected with certain drugs such as AUY922. These results demonstrated that our risk signature could be used for prognosis prediction; additionally, it was also related to the TIME that might act as a potential indicator to instruct immunotherapeutic strategies.

**Conclusion:** This work explored the significance of the risk model constructed by pyroptosis-related lncRNAs in prognosis prediction and its internal link with the immune microenvironment of PAAD. The results are expected to assist in the diagnosis, prognostic assessment, and management of patients with PAAD.

## Introduction

Pancreatic adenocarcinoma [PAAD, a major histological type of pancreatic cancer (PC)] remains an aggressive and lethal solid malignancy despite the developments made in diagnosis and management over the past few years, and traditional treatment strategy still pays little efficiency in improving the prognosis of cancer patients. Cancer statistics showed that the incidence and mortality rate of PC were, respectively, 2.6 and 4.7% around the world, ranked the seventh place in cancer-related deaths, and it was noticeable that the new cases and deaths of PC are almost identical, implying the poor clinical outcome of this devastating disease (Sung et al., 2021). In this era of precision medicine and individualized treatment, numerous explorations have been conducted to investigate the valuable therapeutic targets, although there is no doubt that radical operation remains the only possible curative means for patients with PC. And for patients with advance stage or who cannot tolerate surgery, conventional chemotherapy regimens represented by gemcitabine, as well as the emerging first-line chemotherapy options such as nab-paclitaxel plus gemcitabine and FOLFIRINOX, performed certain effects on improving prognosis (Mizrahi et al., 2020). However, a slight extension of survival is far from satisfying. Recently, immunotherapy (represented by immune checkpoint inhibitors and vaccine therapy) leads a new upsurge in the era of comprehensive cancer treatment and brings glad tidings for terminal cancer patients (Schizas et al., 2020). Regrettably, a major obstacle to cytotoxic or immunological therapeutics is the microenvironment of PC, which possesses a significant desmoplastic characteristic, and this matrix barrier consists of several immunosuppressive cells (like cancer-associated fibroblasts and tumor-associated macrophages) exerts an adverse influence on therapeutic reactivity (Ho et al., 2020). For example, tumor-associated macrophages up-regulated PD-L1 expression in cancer cells by modulating the PKM2 nuclear translocation and exerted an immunosuppressive effect in PAAD (Xia et al., 2022). Cancer-associated fibroblasts impaired immune function by facilitating the expression of co-inhibitory markers on CD4^+^ and CD8^+^ T cells (Gorchs et al., 2019). All these reasons lead to the unsatisfactory response of immunotherapy represented by PD-1 in pancreatic cancer. Therefore, the exploration of novel therapeutic strategies that integrate pancreatic cancer cells and their microenvironment remains critical for the management of pancreatic carcinoma.

Pyroptosis refers to a emerging type of programmed cell death (PCD), which was initiated by gasdermins (represented by GSDMD) through the canonical and non-canonical approaches, with the features of cellular swelling, membrane rupture, and the secretion of cytosolic proinflammatory mediators like IL-1β and IL-18 (He et al., 2015). Pyroptosis is originally regarded as a pivotal regulatory mechanism for anti-infective effectiveness (Jorgensen and Miao, 2015). However, for the past few years, numerous studies have demonstrated that it also assumes a pivotal role in the progression of tumors. It has been reported that the key mediators of pyroptosis such as gasdermin proteins are closely related to tumor development (Sarrió et al., 2021). Meanwhile, the activation of pyroptosis tends to be accompanied by a severe inflammatory response that may affect the TIME; therefore, like other forms of PCDs such as apoptosis, autophagy, and ferroptosis, pyroptosis has become one of the hot topics in current cancer research (Hsu et al., 2021). Recent studies have investigated the prognostic significance of pyroptosis and its connections with the TIME (Ping et al., 2021; Sun et al., 2021; Wu et al., 2021; Ye et al., 2021). However, whether pyroptosis is correlated with the prognosis or specific microenvironment of patients with PAAD or not remains unclear.

Long non-coding RNAs (lncRNAs, > 200 nt in length) refer to a cluster of transcripts originating from the corresponding genes that do not encode proteins. Different from the mRNA, which is responsible for protein translation, although lncRNA does not serve as a messenger between the protein and DNA molecule, it can play an important role in various physiology and pathology processes through a diverse range of mechanisms, like epigenetic and posttranslational modification, transcriptional regulation, as well as RNA–protein stability modulation (Bridges et al., 2021). Not surprisingly, lncRNAs are closely correlated with the generation and progression of malignancies in consideration of their diversified regulatory capabilities (Huarte, 2015).

Recent studies have reported that there is also an intimate connection between the lncRNAs and pyroptosis. For example, lncRNA nuclear paraspeckle assembly transcript 1 (NEAT1) modulated the radiosensitivity of colorectal cancer (CRC) cells by regulating the pyroptosis induced by ionizing radiation through miR-448/GSDME axis (Su et al., 2021). Additionally, lncRNA maternally expressed gene 3 (MEG3) induced pyroptotic cell death in triple-negative breast cancer via MEG3/NLRP3/caspase-1/GSDMD signaling pathway in response to cisplatin treatment, which performed an anti-tumor effect both *in vitro* and *in vivo* (Yan et al., 2021). However, few related studies have been reported in PAAD until now. In our work, we gathered pyroptosis-related lncRNAs (PRlncRNAs) and assessed their connection with the TIME, as well as the possibility to serve as a prospective biomarker in prognosis evaluation of PAAD patients.

## Materials and Methods

### Data Gathering and Processing

The transcriptome dataset, somatic mutation information, and CNV records, as well as the matching clinical data of PAAD patients, were collected from The Cancer Genome Atlas Dataset (TCGA). Perl language was used to transform the raw Ensemble IDs to the corresponding gene symbols based on the annotation information in the Ensemble database. Aiming to normalize the transcriptome data, we converted the initial values of the fragments per kilobase of transcript per million fragments mapped (FPKM) data to transcripts per kilobase million (TPM) values through the R “limma” package (version 3.46.0). After discarding the cases without complete clinical information, finally, we extracted 177 PAAD samples as an integral data cohort for the subsequent analysis.

### Filtration of Prognostic PRlncRNAs

According to the previous research, a total of 52 pyroptosis-related genes (PRGs) were gathered for our exploration (Supplementary Table S1) (Wang et al., 2021; Ding et al., 2022). The lncRNA list was distinguished from the gene expression matrix based on the GTF annotation files by Perl language. Then, the correlation between the lncRNAs and PRGs was performed with Pearson correlation analysis, and PRlncRNAs were filtered out that satisfied the association coefficient |*R* ^2^|>0.4 and *p* <0.001, and these lncRNAs were extracted for the follow-up univariate Cox analysis (*p* <0.05) to identify the prognosis-related PRlncRNAs.

### Unsupervised Clustering Analysis

The prognosis-related PRlncRNAs obtained were subsequently applied in non-negative matrix factorization (NMF) clustering analysis using the R “NMF” package (version 0.23.0). The accuracy of clustering was guaranteed through 10 iterations (rank = 2–10). Then, 177 PAAD patients were divided into different subtypes based on this analysis and were carried out for further data mining. Analysis of survival differences between specific clusters was conducted through the Kaplan–Meier (K-M) survival analysis, and the result was visualized using the R “survminer” package (version 0.4.9); the differences in clinicopathological features were also displayed in a heatmap using the R “pheatmap” package (version 1.0.12). Gene set variation analysis (GSVA) was performed to explore the potential biological pathways between different clusters. Moreover, CIBERSORT is an algorithm for assessing the variation in immune cell infiltration among different subgroups, and the ESTIMATE algorithm is usually applied to score the degree of immune/stromal infiltration in tumor samples, both algorithms were performed to reveal the immune infiltration signature of the PRlncRNAs-related clusters in our work.

### Exploitation and Validation of PRlncRNAs Based Prognostic Model

Lasso (least absolute shrinkage and selection operator) Cox regression analysis was utilized to establish a prognostic model based on the univariate Cox result. First, to enhance the dependability of the prediction model, the entire dataset involving 177 patients was randomized to a training cohort (*n* = 89) and a testing cohort (*n* = 88) through the R “caret” package (version 6.0–90) (Deist et al., 2018), and then lasso analysis was operated in both training and validation set using the R “glmnet” package (version 4.1–3). The risk score of each case was calculated according to the formula below: Risk score = ΣCoef PRlncRNAs × Exp PRlncRNAs (Coef: coefficient of each PRlncRNA, Exp: expression of each PRlncRNA), and on the basis of the median risk score, the whole cases were distributed into high/low-risk subgroups. In order to evaluate the performance of this risk signature in prognosis prediction of PAAD patients, we further investigated the predictive ability in the training, validation, and the whole set with the help of K-M survival curves and time-dependent receiver operating characteristic (ROC) curves generated by the R “suvminer” and “time ROC” packages (version 0.4). The PRlncRNAs expression levels between the high/low-risk groups and the survival situations of patients at different risk scores were exhibited with heatmap and scatter chart more intuitively. Additionally, the connections among risk scores, clinical characteristics, clusters, as well as immune scores were also explored.

### Functional Enrichment Analysis

Gene set enrichment analysis (GSEA) was used to investigate potential enrichment pathways or biological functions based on the differentially expressed gene sets among different subgroups. Here, we conducted GSEA analysis to explore the latent biological pathways of PRlncRNAs between the high/low-risk groups according to the predefined gene sets (c2. cp.kegg.v7.4. symbols.gmt). Additionally, the differentially changed genes of the two risk groups [|log (foldchange)|>1, FDR<0.05] were also extracted, and then Gene Ontology (GO) and Kyoto Encyclopedia of Genes and Genomes (KEGG) enrichment analysis were executed with the R “clusterProfiler” package (*p* <0.05).

### Investigation of Immune Infiltration Signature

In this section, we combined multiple immune infiltration assessment methods besides the CIBERSORT algorithm, such as CIBERSORT-ABS, TIMER, QUANTISEQ, MCPCOUNTER, XCELL, and EPIC, to reveal the differences in the immune microenvironment between the two risk groups more comprehensively. Also, we employed single-sample gene set enrichment analysis (ssGSEA) to estimate the immune function of high/low-risk groups through the R “GSVA” package (version 1.38.2). And the discrepancies in immune checkpoint expression levels were analyzed based on the 47 related genes to foretell the therapeutic response to immune checkpoint inhibitors (ICIs) in both risk groups. Furthermore, correlations between PRlncRNAs and infiltrated immune cells as well as PRGs were analyzed by Spearman association analysis and visualized with heatmaps.

### Construction of Tumor Mutation Signature

Somatic mutation landscape was described with waterfall plot based on the R “maftools” package, and the first 20 genes with the most frequency of alteration as well as their mutation types in both risk groups were displayed respectively. Tumor mutation burden (TMB) means the total amount of somatic mutation investigated per million bases except for the germline mutation, including the errors of somatic gene coding, base substitutions, and the mistakes of gene insertion or deletion. Some studies have indicated that TMB is related to cancer survival and immunoreactivity, which can act as a predictor of the curative effects of immunotherapy; patients with a high-TMB score tend to benefit from immunological therapeutics (Goodman et al., 2017; Klebanov et al., 2019). Therefore, we investigated the association between TMB and risk scores as well as their impacts on patients’ survival, and the connections among TMB, risk scores, and immune cells infiltration were also shown with a chord diagram based on the R “circlize” package (version 0.4.13).

### Drug Susceptibility Analysis

Aiming to discover the possible sensitive drugs in high/low-risk groups, we employed the R “ggplot2” and “pRRophetic” packages to count the lower half inhibitory concentration (IC50) of frequently used anti-cancer agents according to the gene expression of PAAD cases (Geeleher et al., 2014).

### Statistical Analysis

Data statistical analysis involved in this work was accomplished with the R software (version 4.0.4). The mutation landscape of 52 PRGs was displayed with a waterfall plot based on the “maftools” package, and the CNV feature of these PRGs and their location on 23 pairs of chromosomes were also analyzed based on the “RCircos” package. Differential expression of both PRGs and prognostic PRlncRNAs was identified using the “limma” package. The Chi-square test was used to compare whether or not there were significant differences in clinicopathologic features between the two risk groups. For each analysis, *p* <0.05 was considered as the threshold value of significance.

## Results

### Genetic Variation Landscape of 52 Pyroptosis-Related Genes

**FIGURE 1:**
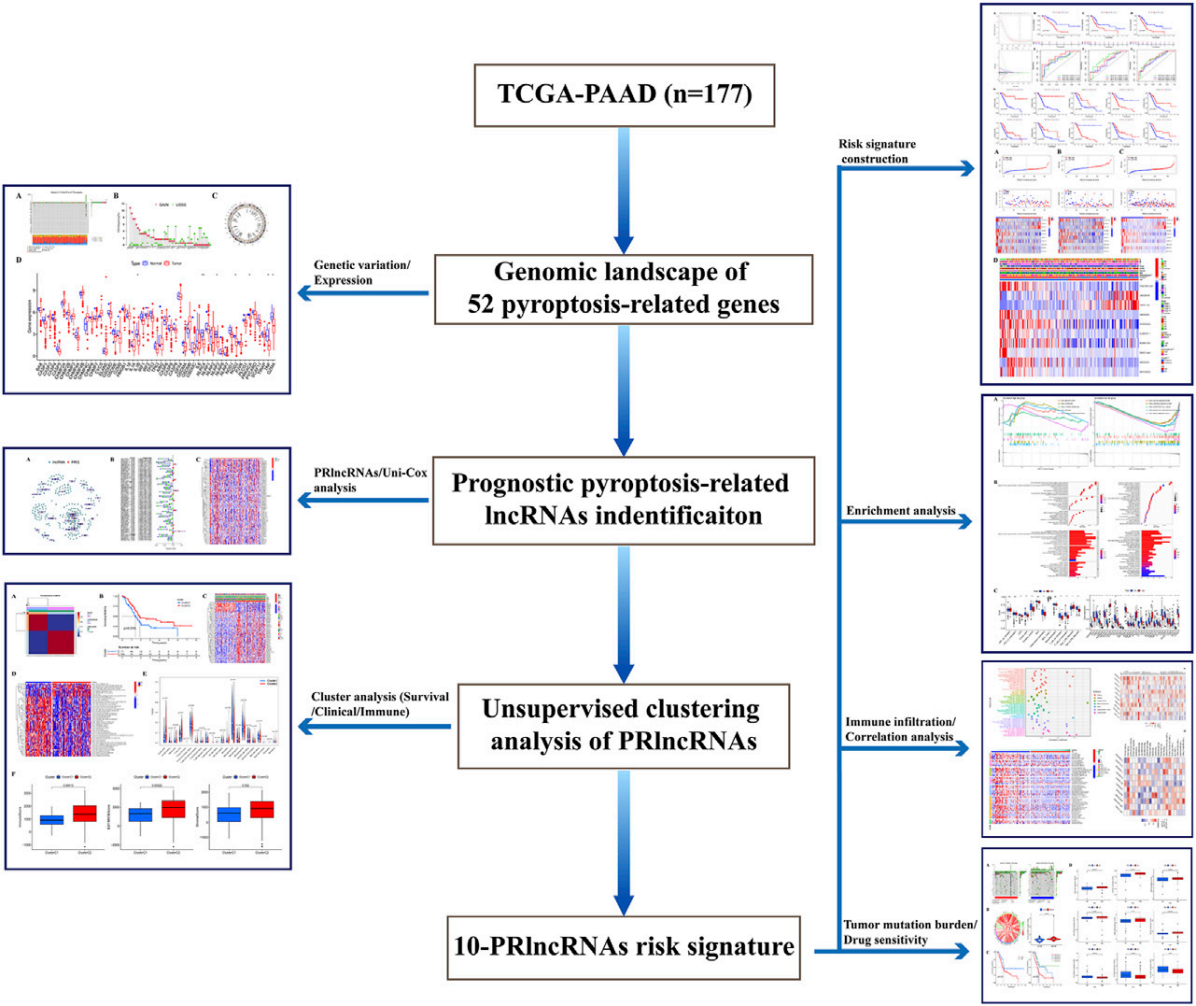
Flow chart of this work.

**FIGURE 2:**
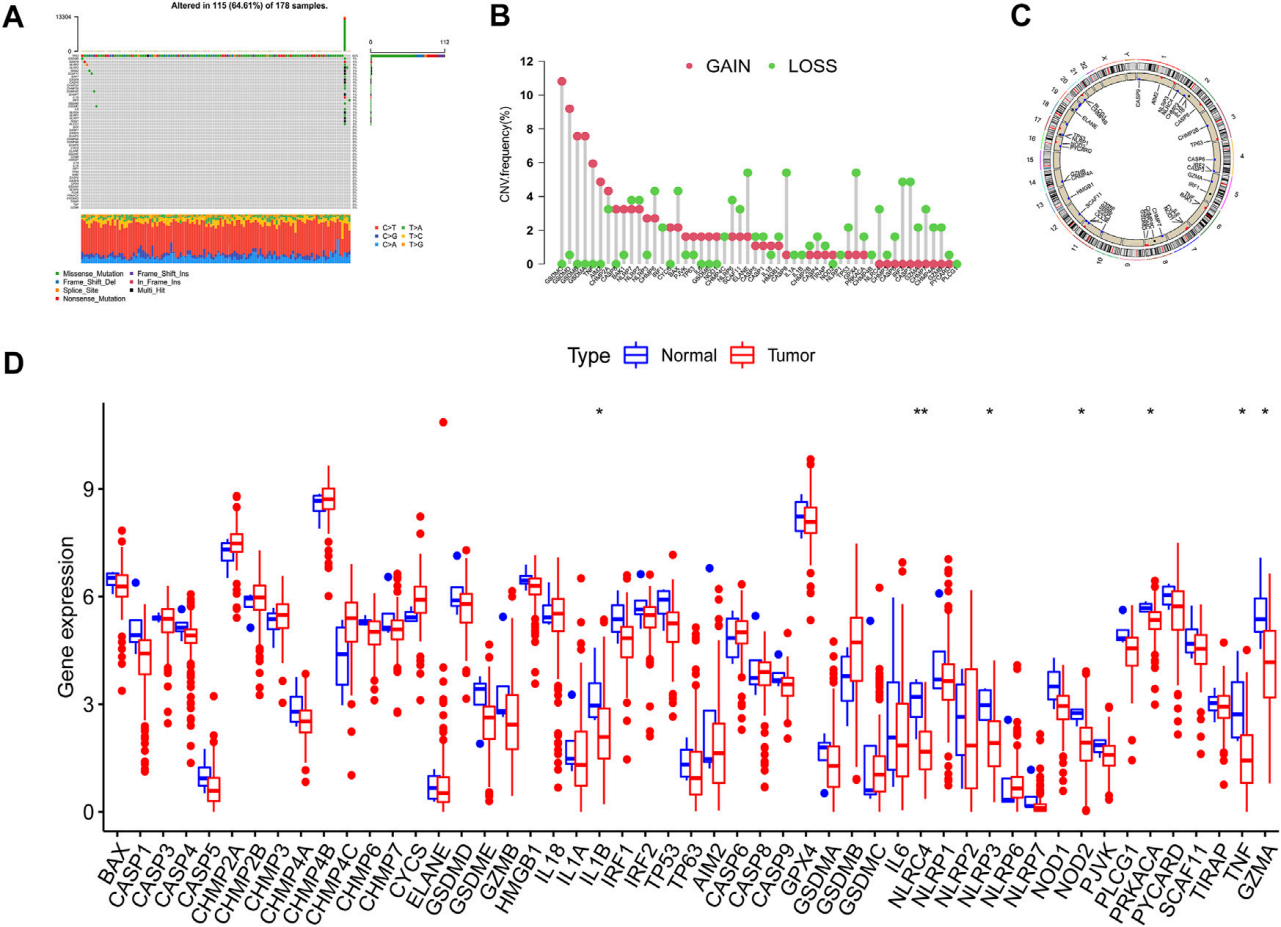
Variation feature of 52 pyroptosis-related genes (PRGs) in pancreatic adenocarcinoma (PAAD). **(A)** Somatic mutation statistics: The overall mutation frequency was 64.61% (115 in 178 samples), TP53 accounted for the highest alteration frequency, and missense mutation was the predominant alternation type. **(B)** Copy number variation (CNV) analysis: CNV alteration was common among these PRGs, GSDMC, GSDMD, GSDMB, and GSDMA performed with CNV gain primarily, ELANE, CASP9, and GPX4 mainly presented with CNV loss. **(C)** Chromosomal location of PRGs. **(D)** The expression level of PRGs in normal and neoplastic samples was displayed by boxplot (*p < 0.05; **p < 0.01).

The overall flow diagram of this work was exhibited in Figure 1. As Figure 2A showed that 115 among 178 PAAD cases underwent somatic mutations with a frequency of 64.61%, TP53 accounted for the highest alteration frequency, and the missense mutation was the most common mutation type. The CNV changes in 52 PRGs were also analyzed and the results revealed that CNV alteration was a common occurrence among these PRGs. Among these CNV changes, GSDMC, GSDMD, GSDMB, and GSDMA had a high frequency of CNV gain, while ELANE, CASP9, and GPX4 mainly presented with CNV loss (Figure 2B). In addition, Figure 2C described the position of the CNV changes in these PRGs on the chromosomes. And the differential expression of 52 PRGs was shown in Figure 2D.

### Identification of Pyroptosis-Related lncRNAs

**FIGURE 3:**
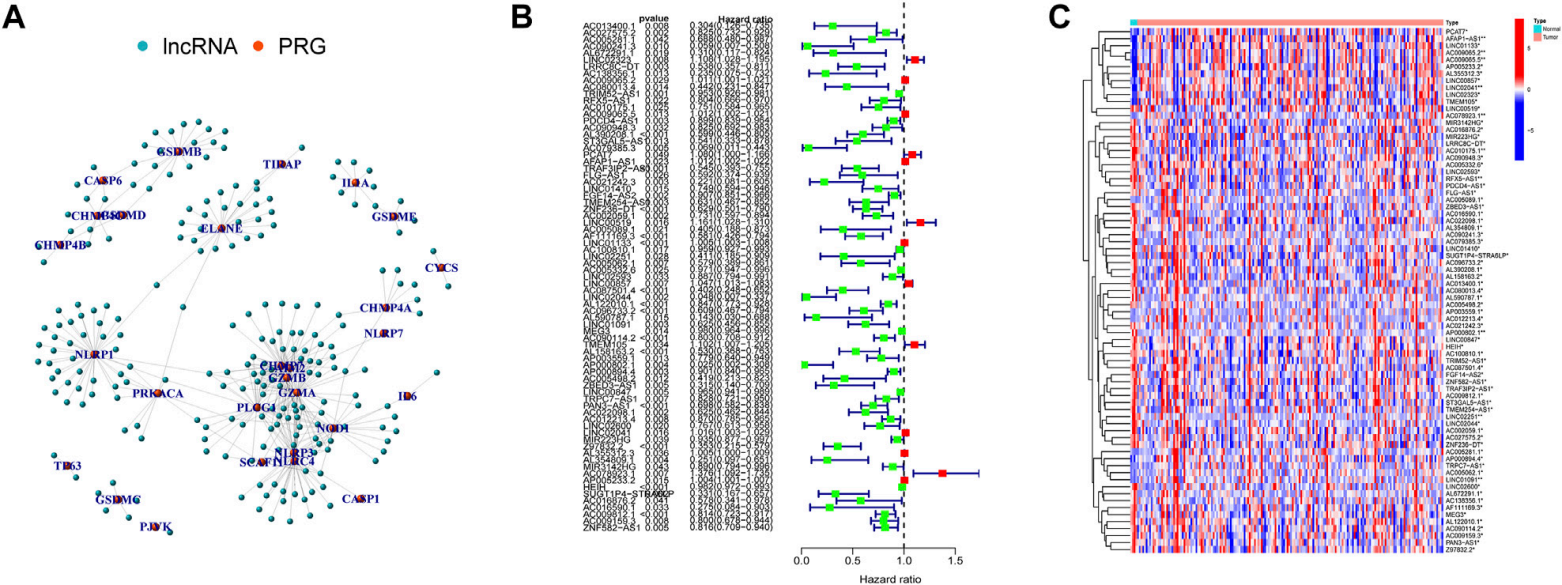
Pyroptosis-related lncRNAs (PRlncRNAs) identification. **(A)** A total of 226 PRlncRNAs correlation network. **(B)** Univariate Cox analysis was conducted to gain prognosis-related PRlncRNAs. **(C)** Heatmap showed the differential expression of 75 prognostic PRlncRNAs (*p < 0.05; **p < 0.01).

Here, 226 PRlncRNAs were identified through the Pearson correlation analysis, and their linked network was displayed in Figure 3A. Furthermore, 75 prognostic PRlncRNAs were extracted using univariate Cox analysis (Figure 3B, Supplementary Table S2), and their expression levels were displayed in Figure 3C.

### Clustering and Bioinformatic Analysis

**FIGURE 4:**
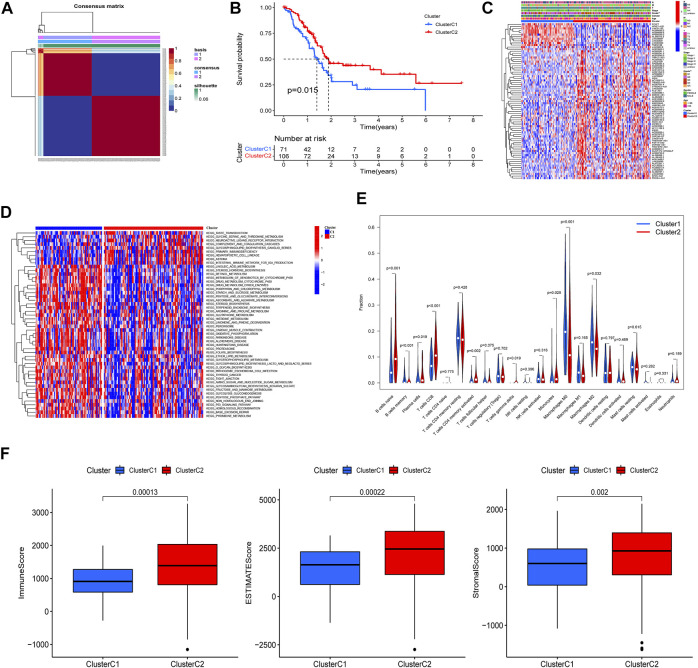
PAAD cluster analysis based on prognostic PRlncRNAs. **(A)** A total of 177 PAAD patients were classified into two clusters through the unsupervised cluster analysis (Cluster C1: 71, Cluster C2: 106). **(B)** K-M survival curve showed that cluster C2 had a poorer prognosis compared to cluster C1 (*p* = 0.015). **(C)** The relationship between clinicopathological features and clusters was shown in the heatmap (**p < 0.01). **(D)** Gene set variation analysis (GSVA) revealed different biological pathways between the two clusters. **(E)** The violin plot displayed the differences in immune cell infiltration between two clusters according to the CIBERSORT algorithm. **(F)** Immune, Estimate, and Stromal scores were calculated in two clusters through the ESTIMATE algorithm, and all three achieved higher scores in cluster C2.

Based on the 75 prognostic PRlncRNAs, we performed an unsupervised clustering analysis to classify patients through the “NMF” R package. A total of 177 PAAD patients with complete survival information were divided into two different clusters (rank = 2), 71 in cluster C1 and 106 in cluster C2 (Figure 4A); the intergroup correlation was minimal while the intragroup connection was significant under such condition (Supplementary Figure S1). And the differences in the PRGs between the patient subtypes identified by PRlncRNAs were also shown in Supplementary Figure S1. The survival curves demonstrated that cluster C2 had a better prognosis compared to cluster C1 (Figure 4B). Subsequently, we analyzed the correlation between clinicopathological features and clusters, and the tumor grade displayed a significant difference between these two specific clusters (Figure 4C). And gene set variation analysis investigated that cluster C1 was mainly enriched in metabolism-related pathways such as linoleic acid metabolism, retinol metabolism, and drug metabolism, which was linked to some nervous system diseases like Parkinson’s, Alzheimer’s, and Huntington’s disease, while cluster C2 was correlated with immune-related pathways like primary immunodeficiency and intestinal immune network for IGA production (Figure 4D). Moreover, the infiltrated fractions of immune cells had also been calculated according to the CIBERSORT algorithm, and higher infiltration of naïve B cells, plasma cells, CD8-T cells, CD4 memory-activated T cells, gamma–delta T cells, and monocytes were observed in cluster C2, while the infiltrated levels of memory B cells and macrophages (M0 and M2 subtype) were much higher in cluster C1 (Figure 4E). Figure 4F reflected the discrepancy of immune function between the two clusters based on the ESITMATE algorithm; compared to cluster C1, cluster C2 had notably higher immune scores, indicating that the immune status of cluster C2 might be a key factor contributing to its survival benefit.

### Construction and Verification of PRlncRNAs-Related Risk Signature

**FIGURE 5:**
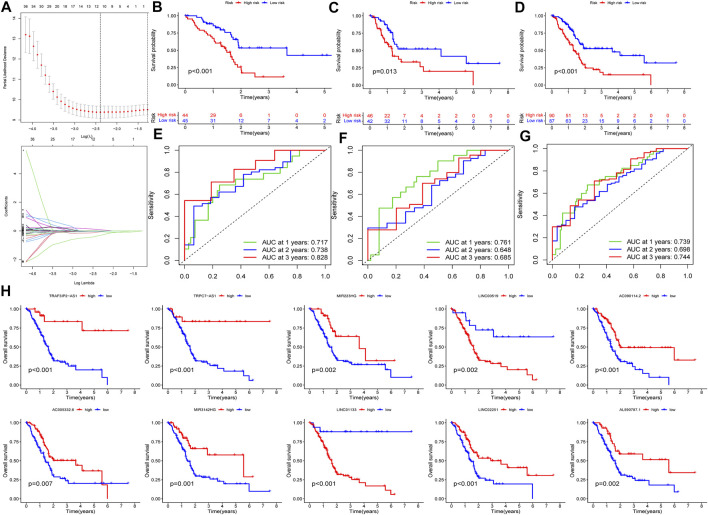
Construction and validation of PRlncRNAs risk model. **(A)** Lasso Cox regression analysis. **(B–D)** K-M survival curves of patients in the training, testing, and entire dataset. **(E–G)** Receiver operating characteristic (ROC) curves of three cohorts. **(H)** Independent prognostic analysis of 10 PRlncRNA risks.

**FIGURE 6:**
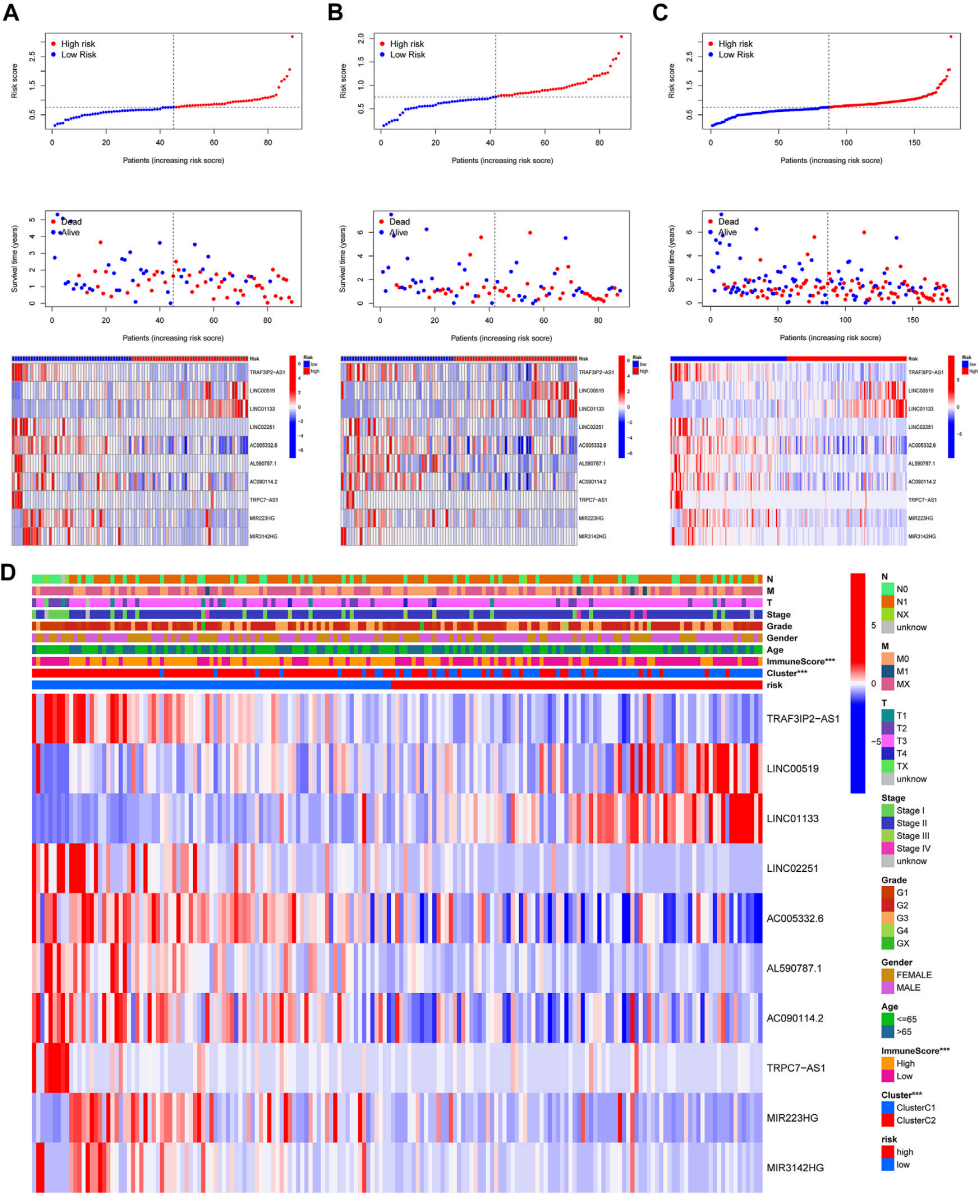
Risk survival status and the connection among risk scores, clusters, and clinical features. **(A–C)** Patients’ survival conditions in the training set (left), testing set (middle), and entire dataset (right): risk scores (upper), survival status (middle), and PRlncRNAs levels (below). **(D)** The connection of the risk score with the clinical characteristics and clusters (***p < 0.001).

In this part, 177 PAAD cases were randomized to a training cohort (*n* = 89) and a validation cohort (*n* = 88) firstly (Table 1). Then, we conducted lasso Cox regression analysis using the 75 PRlncRNAs with prognostic values in univariate Cox analysis. And 10 representative PRlncRNAs were filtered out to establish risk models, including TRAF3IP2-AS1, LINC00519, LINC01133, LINC02251, AC005332.6, AL590787.1, AC090114.2, TRPC7-AS1, MIR223HG, and MIR3142HG. Furthermore, we counted the risk score of each sample utilizing both lasso regression coefficient and expression of each PRlncRNA, according to the formula below: Risk score = (−0.040762063483424 × expressionTRAF3IP2-AS1) + (0.0989793438963536 × expressionLINC00519) + (0.00250080194225471 × expressionLINC01133) + (−0.355405520488876 × expressionLINC02251) + (−0.00678548407679974 × expressionAC005332.6) + (−0.0909344568017417 × expressionAL590787.1) + (−0.0408541524198621 × expressionAC090114.2) + (−0.0181443731301019 × expressionTRPC7-AS1) + (−0.00474532268973943 × expressionMIR223HG) + (−0.0423278887877701 × expressionMIR3142HG) (Figure5A; Table 2). And patients in both cohorts were grouped according to the median score for the subsequent study (high/low-risk group). Next, we assessed the value of this risk signature involving 10 lncRNAs in prognostic prediction. Survival analysis found that patients with high-risk scores suffered a worse prognosis than those low-risk ones in the training cohort (Figure 5B); this result had also been validated both in the testing cohort and entire cohort (Figures 5C,D). Additionally, ROC curves exhibited that the aera under curves (AUC) of 1, 2, and 3 years in the training set were 0.717, 0.738, and 0.828, and 0.761, 0.648, 0.685 in the testing set, 0.739, 0.698, 0.744 in the entire dataset, respectively, indicating that our risk model possessed a certain accuracy to serve as a prognostic prediction tool for PAAD patients (Figures 5E–G). Specifically, independent prognostic analysis of these 10 lncRNAs showed that patients with higher expression levels of LINC01133 and LINC00519 had a worse prognosis while lower levels of the other 8 lncRNAs related to a poorer overall survival (Figure 5H). And we also investigated the impacts of risk scores on survival in patients with different clinicopathologic features; the results showed that tumor grade (G1–2), stage (Stage I–II), and TN staging were closely related to survival (Supplementary Figure S3). Figures 6A–C described the survival situation of patients at high- and low-risk scores, and the result found that death cases increased with the increase in risk scores, suggesting that our models can distinguish PAAD patients well, and the heatmap also painted the differential expression patterns of 10 lncRNAs between the high/low-risk groups; except for LINC01133 and LINC00519, the rest of lncRNAs expressed a higher level in the low-risk group. Moreover, we also investigated the connection among clinical characteristics, clusters, immune scores, and risk scores, the results manifested that risk scores were markedly correlated with clusters and tumor immune scores (Figure 6D).

**TABLE 1:** Clinicopathological information of PAAD patients in the training, testing, and entire cohort.

Covariates		Entire cohort	Training cohort (*n* = 89)	Testing cohort (*n* = 88)	*p*-value
Age					
<=65		93 (52.54%)	51 (57.3%)	42 (47.73%)	0.2605
>65		84 (47.46%)	38 (42.7%)	46 (52.27%)	
Gender					
Female		80 (45.2%)	37 (41.57%)	43 (48.86%)	0.4103
Male		97 (54.8%)	52 (58.43%)	45 (51.14%)	
Grade					
G1		31 (17.51%)	17 (19.1%)	14 (15.91%)	0.5497
G2		94 (53.11%)	51 (57.3%)	43 (48.86%)	
G3		48 (27.12%)	19 (21.35%)	29 (32.95%)	
G4		2 (1.13%)	1 (1.12%)	1 (1.14%)	
GX		2 (1.13%)	1 (1.12%)	1 (1.14%)	
Stage					
I		21 (11.86%)	9 (10.11%)	12 (13.64%)	0.6233
II		146 (82.49%)	73 (82.02%)	73 (82.95%)	
III		3 (1.69%)	2 (2.25%)	1 (1.14%)	
IV		4 (2.26%)	3 (3.37%)	1 (1.14%)	
Unknown		3 (1.69%)	2 (2.25%)	1 (1.14%)	
T stage					
T1		7 (3.95%)	3 (3.37%)	4 (4.55%)	0.8296
T2		24 (13.56%)	12 (13.48%)	12 (13.64%)	
T3		141 (79.66%)	70 (78.65%)	71 (80.68%)	
T4		3 (1.69%)	2 (2.25%)	1 (1.14%)	
TX		1 (0.56%)	1 (1.12%)	0 (0%)	
Unknown		1 (0.56%)	1 (1.12%)	0 (0%)	
M stage					
M0		79 (44.63%)	38 (42.7%)	41 (46.59%)	0.5625
M1		4 (2.26%)	3 (3.37%)	1 (1.14%)	
MX		94 (53.11%)	48 (53.93%)	46 (52.27%)	
N stage					
N0		49 (27.68%)	25 (28.09%)	24 (27.27%)	0.6047
N1		123 (69.49%)	61 (68.54%)	62 (70.45%)	
NX		4 (2.26%)	3 (3.37%)	1 (1.14%)	
Unknown		1 (0.56%)	0 (0%)	1 (1.14%)	

**TABLE 2:** Ten PRlncRNAs extracted by lasso regression.

Lasso lncRNAs	Coefficient
TRAF3IP2-AS1	−0.040762063
LINC00519	0.098979344
LINC01133	0.002500802
LINC02251	−0.35540552
AC005332.6	−0.006785484
AL590787.1	−0.090934457
AC090114.2	−0.040854152
TRPC7-AS1	−0.018144373
MIR223HG	−0.004745323
MIR3142HG	−0.042327889

### Functional Enrichment Analysis of the Risk Models

**FIGURE 7:**
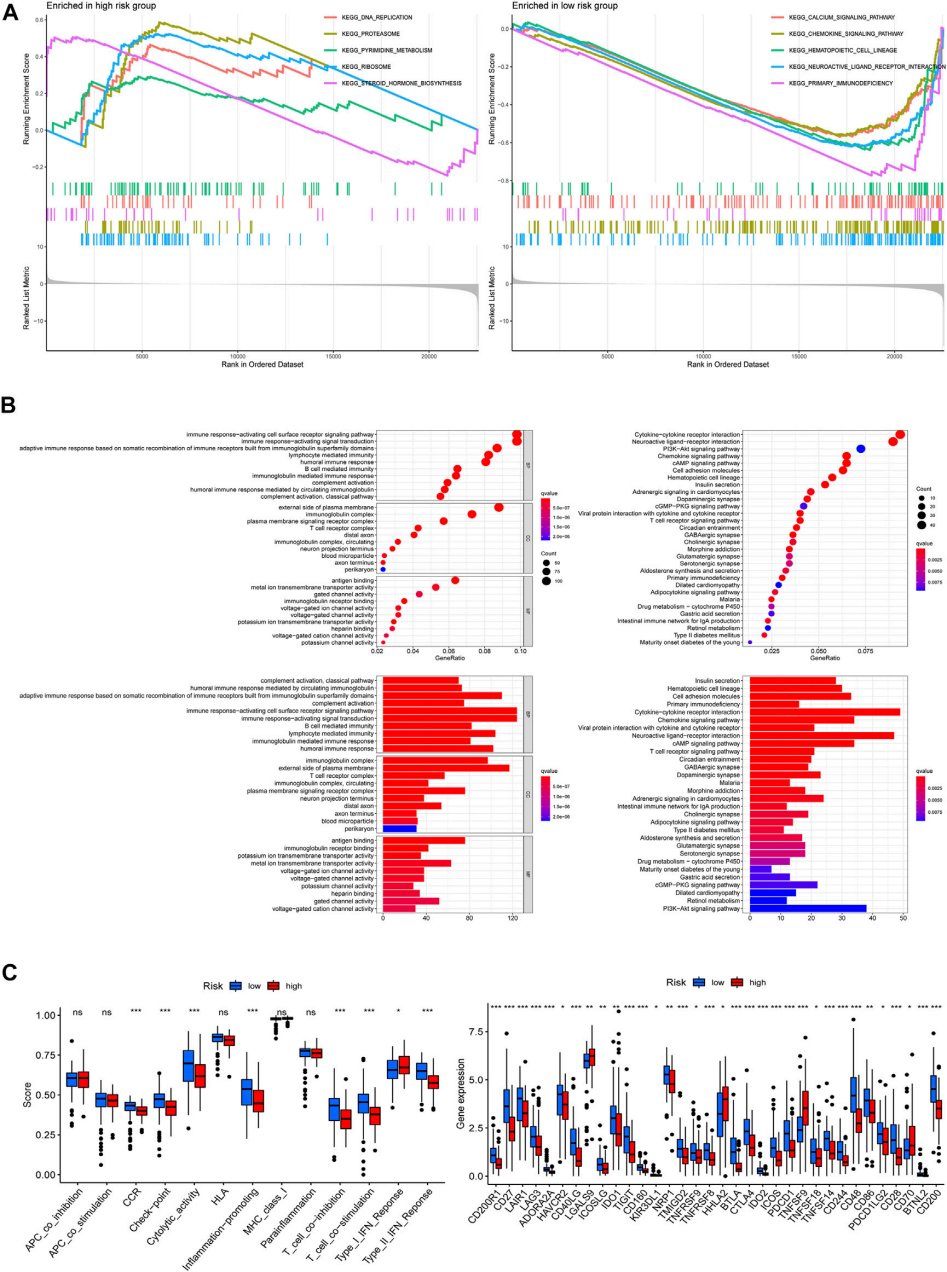
Functional enrichment analysis. **(A)** Gene set enrichment analysis (GSEA) in both risk groups. **(B)** Differentially expressed genes between the two groups: Gene ontology (GO) enrichment analysis (left) and Kyoto Encyclopedia of Genes and Genomes (KEGG) pathway enrichment analysis (right). **(C)** Immune functions analysis: immune function scores between the two risk groups (left), expression patterns of immune checkpoint genes (right) (*p < 0.05; **p < 0.01; ***p < 0.001; ns, not significant).

To investigate the latent functional pathways, we employed GSEA in two risk groups respectively. Here, we listed the top 5 enrichment pathways for each risk group; as the results showed, DNA replication, proteasome, ribosome, steroid hormone biosynthesis, and pyrimidine metabolism signaling pathways were mainly enriched in the high-risk group, while the low-risk group was concentrated in calcium, chemokine, hematopoietic cell lineage, neuroactive ligand–receptor interaction, and primary immunodeficiency pathways (Figure 7A). To further investigate the differences in biological functions and pathways between the two risk groups, we conducted GO and KEGG analysis using the differential genes between the high/low-risk groups. The results of KEGG analysis showed that the differential genes were mainly enriched in some receptor–ligand interaction-related pathways, like cytokine–cytokine receptor interaction, neuroactive ligand–receptor interaction, as well as chemokine signaling pathways. GO enrichment analysis indicated that the differential genes mainly concentrated in immune-related biological processes, including immune response-activating cell surface receptor signaling pathway and immune response-activating signal transduction (Figure 7B). And Figure 7C displayed the differences in immune functions and immune checkpoint genes (ICGs) between the two groups, the results indicated that the score of immune functions (like check-point, inflammation promoting, T-cell co-inhibition, and co-stimulation) had a remarkable difference between the two risk groups, and nearly all of ICGs expressed a higher level in patients at low-risk scores, implying that the risk signature which we built might be closely related to patient’s immune response.

### Correlation Evaluation Between Risk Score and Immune Infiltration

**FIGURE 8:**
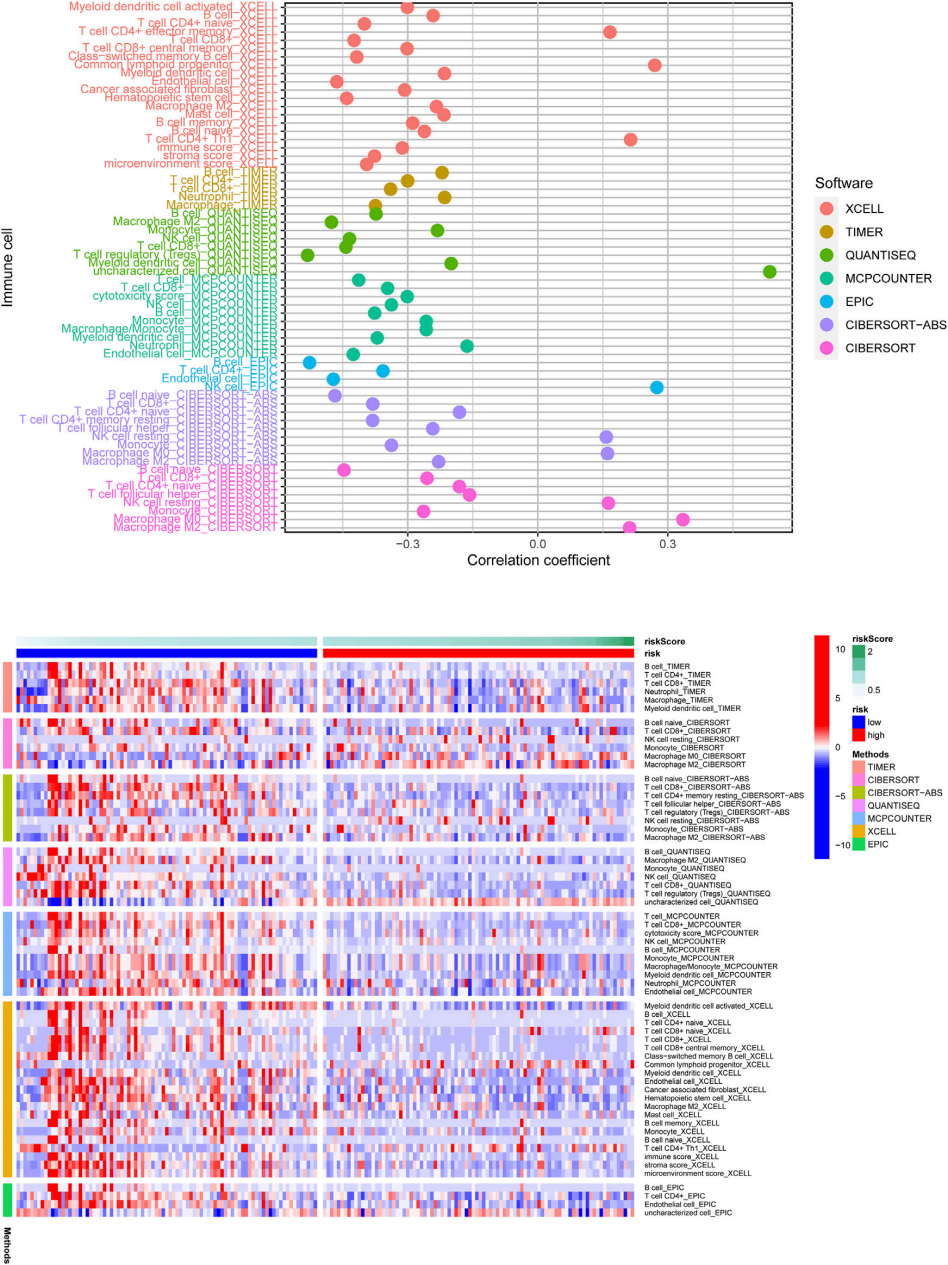
The correlation of risk scores and immune cells infiltration was analyzed through multiple immune infiltration algorithms, and the result was shown with the lollipop plot (upper) and heatmap (below). Most immune cells were negatively related to the risk score observed from the result.

**FIGURE 9:**
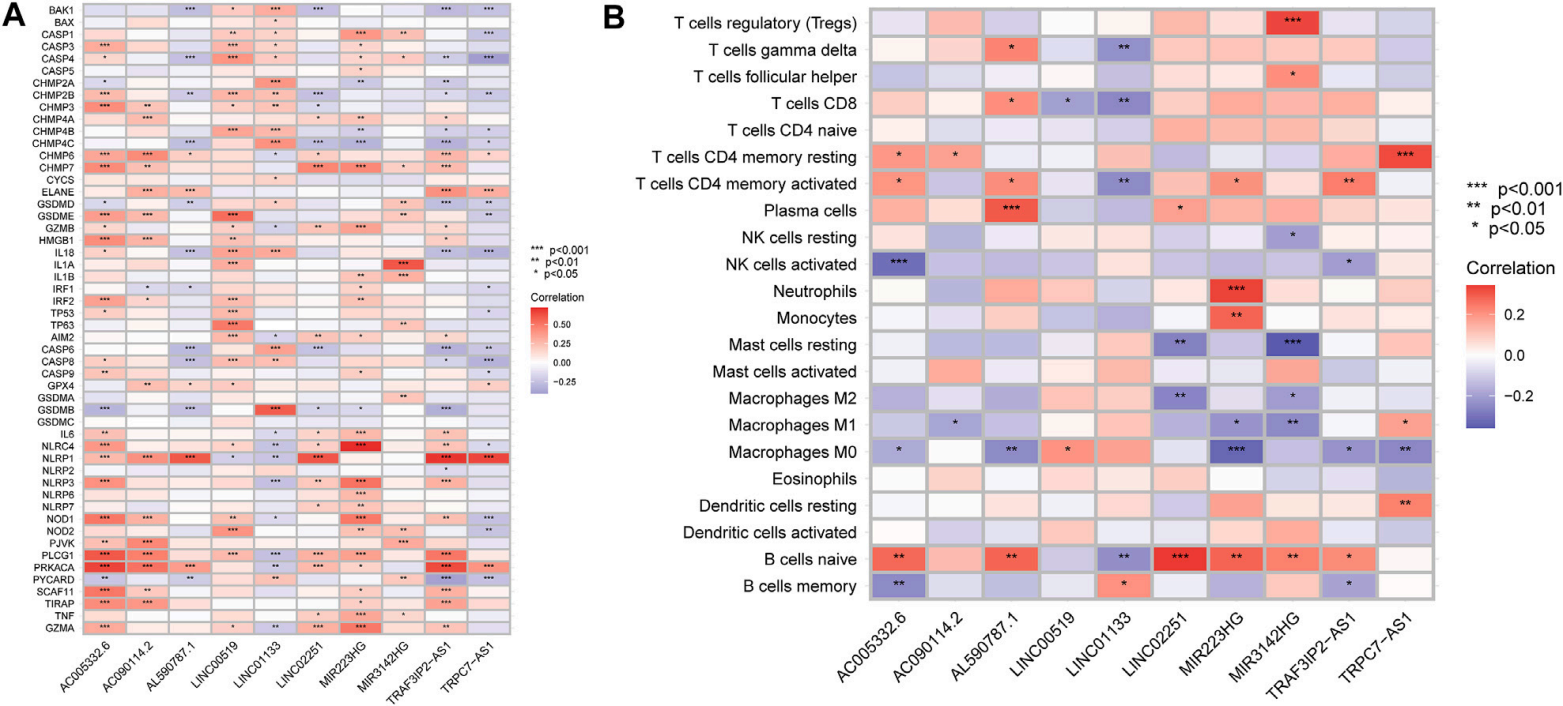
Correlation analysis. **(A)** Correlation of PRlncRNAs and PRGs. **(B)** Correlation of PRlncRNAs and immune cells. Red refers to positive relation while blue refers to negative relation (*p < 0.05; **p < 0.01; ***p < 0.001).

According to the above findings, we subsequently investigated the relationship between high/low-risk scores and immune cells infiltration, and as the result displayed in Figure 8, a significant negative connection between immune cells infiltration and risk scores was revealed with the help of several different immune infiltration algorithms; most immune cells (such as CD4^+^ T cells, CD8^+^ T cells, B cells naïve, and NK cells) showed a higher infiltrated abundance in patients with low-risk scores than the high-risk ones. Additionally, correlations between PRlncRNAs and immune cells as well as PRGs were also shown in Figures 9A,B, and we found that there were significant positive correlations between lncRNAs and immune cells, like LINC02251 and B cells naïve, MIR223HG and Tregs, as well as TRPC7-AS1 and T cells CD4 memory resting. Additionally, we further investigated the correlation between PRlncRNAs and PRGs as well as immunomarkers in an external independent dataset [PACA-CA cohort from the International Cancer Genome Consortium (ICGC) database], and the results validated that there was indeed an intricate and tight association between lncRNAs and these genes (Supplementary Figure S4). Interestingly, we observed that the abundance of immune cells infiltration exhibited a high degree of consistency with the expression level of PRlncRNAs between the high/low-risk groups, which was also consistent with the result of our functional enrichment analysis that the low-risk group was concentrated in immune-related pathways, indicating that patients at a low-risk score might be associated with higher immune activity and better prognosis, and this might be attributed to the fact that PRlncRNAs-mediated inflammatory cell death increased immune cells infiltration levels in the microenvironment. While certain immune cells like macrophages (M0, M1, and M2 subtypes) showed a higher infiltration level in patients with high-risk scores numerous studies have demonstrated that macrophages (especially tumor-associated macrophages) act as an important moderator in tumor progression by mediating tumorigenesis and metastasis. So, here our results also provided a new perspective for exploring the internal connection between macrophages and the progression of PAAD.

### Tumor Mutation Burden and Drug Sensitivity Analysis

**FIGURE 10:**
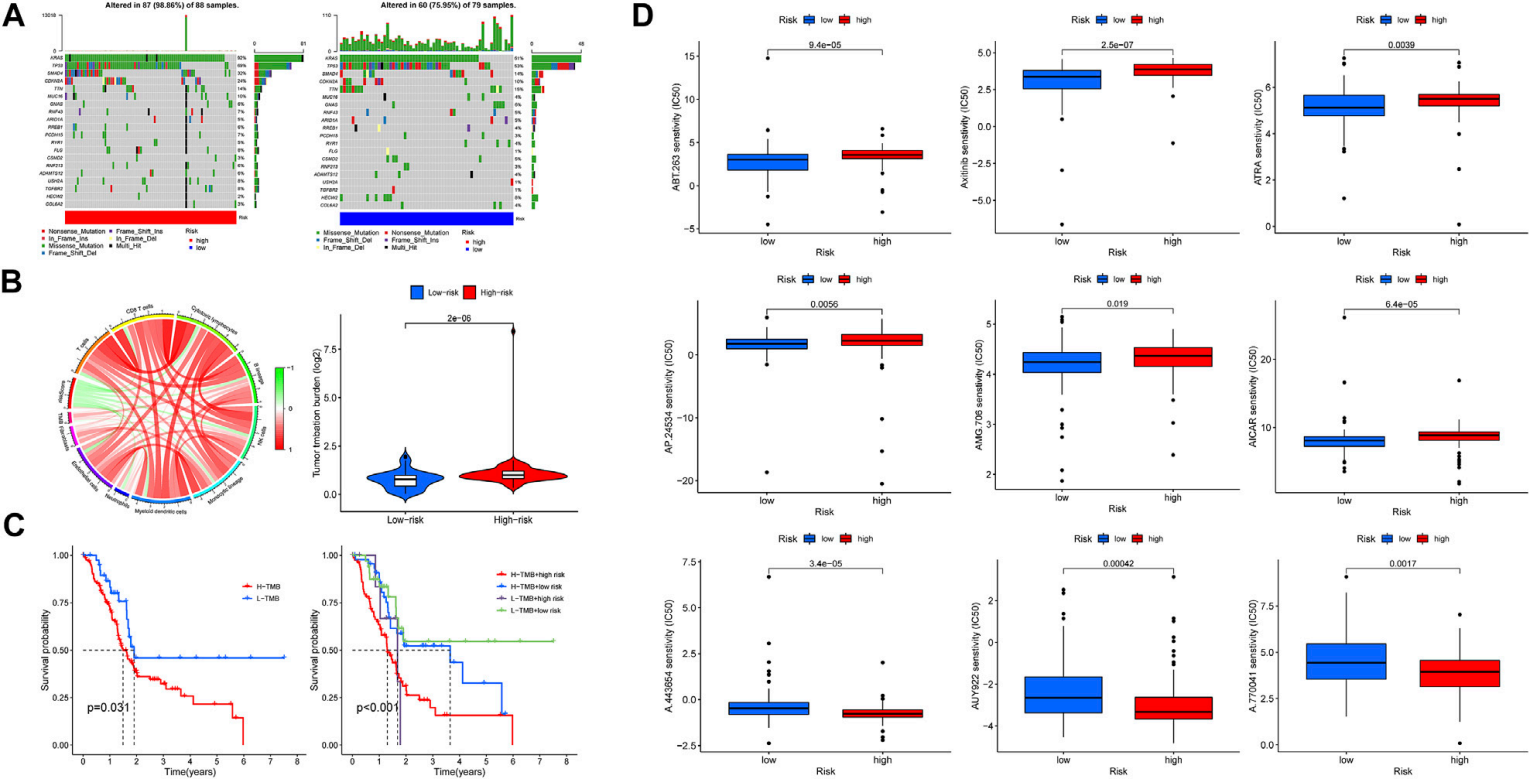
Tumor mutation burden (TMB) and drug sensitivity analysis. **(A)** Somatic mutation landscape of the high- (left) and low-risk (right) groups. **(B)** Association network of risk scores, TMB, and immune cells. Red refers to positive relation, whereas green refers to negative relation. **(C)** K-M survival curve of patients at high/low TMB (left), as well as TMB united with risk scores (right). **(D)** Lower half inhibitory concentration (IC50) of chemotherapeutic agents: the low-risk score was associated with AICAR and Axitinib, while the high-risk score was associated with AUY922.

In this section, we first investigated the tumor mutation landscape of the risk model and its correlation with the survival of PAAD cases. As the results showed, patients with high-risk scores suffered a higher possibility of genetic mutations, KRAS, TP53, SMAD4, CDKN2A, and TTN were the first five genes with the uppermost mutation frequency both in the high/low-risk groups, and the differences of tumor mutation patterns were also exhibited in Figure 10A. Meanwhile, the connections among TMB, immune cells, and risk scores were also explored; the result found that a high-risk score was associated with a high TMB (Figure 10B), survival analysis showed a poorer overall survival in high-TMB patients (*p* = 0.031), and it was noteworthy that patients with both high-TMB and high-risk scores suffered a worse prognosis compared with those at low-TMB or low-risk scores (*p* <0.001) (Figure 10C). These findings suggest that our risk model is markedly related to the TMB of PAAD, the combination of risk scores, and TMB is helpful for prognosis prediction of patients with pancreatic cancer. Based on the findings stated above, we subsequently explored whether risk scores could be able to guide patients’ chemotherapy regiments, and the results indicated that patients with low-risk scores were more responsive to certain chemotherapeutic agents such as AICAR (namely Acadesine, *p* < 0.001) and Axitinib (*p* < 0.001), whereas patients with a high-risk score might respond to agents like AUY922 (namely Luminespib, *p* < 0.001), meaning that our risk signature was able to serve as an indicator for the prediction of chemosensitivity (Figure 10D).

## Discussion

Like other common gastrointestinal carcinomas, timely diagnosis remains a major challenge in the management of PC due to its occult peculiarity in the early stage. The epidemiological investigation found that PC occupied the fourth place in deaths caused by cancers both in the United States and European countries (Ferlay et al., 2018; Siegel et al., 2020), which also acted as the sixth leading contributor to cancer-related deaths in China (Chen et al., 2016), and its burden has been more than doubled over the past few decades globally (Klein, 2021). Despite considerable efforts being made to make a clear awareness of the pivotal risk factors (including smoking, excessive alcohol consumption, and chronic health problems like obesity and diabetes) and explore the available treatment strategies, the 5-year survival rate of PC is still barely 9% which is frustrating (Ferlay et al., 2018; Siegel et al., 2020); therefore, exploration for new credible diagnostic and therapeutic methods is urgently needed at present.

Pyroptosis represents an emerging type of PCDs, which is mainly triggered by the caspase-reliant immunoreactions in response to the pathogen-associated molecular patterns (PAMPs), damage-associated molecular patterns (DAMPs), as well as lipopolysaccharides in the cytoderm of Gram-negative bacterium, including the classical pathway (caspase-1-dependent) and the non-classical pathway (caspase-4/5 or caspase-11-dependent) (Wang et al., 2019). Despite the fact that ICIs and other immunotherapies have revolutionized cancer treatment, there are still limitations to overcome. A key prerequisite for these therapies to exert a steady effect is that the tumor is immune abundant, and CD8+T cells occupy a dominating place in this process (Ferlay et al., 2018; Siegel et al., 2020). Interestingly, pyroptotic cell death has been demonstrated to enhance the anti-tumor effects by altering the immune microenvironment. The essential point is that inflammatory cell death effectively recruits a variety of lymphocytes (including CD8+T cells) and macrophages into the tumor microenvironment, which successfully ignite the immune environment to convert immunologically insensitive tumors into sensitive ones, and this phenomenon is attributed to the secretion of inflammatory cytokines (like IL18, IL1β, ATP, and HMGB1) caused by pyroptosis (Tang et al., 2020; Rosenbaum et al., 2021). For example, sorafenib could motivate the cytotoxicity of natural killer cells (NKs) to hepatocellular carcinoma (HCC) cells by inducing the pyroptotic cell death in macrophages, and this effect was significantly related to the secretion of IL-18 and IL-1β (Hage et al., 2019). HMGB1, released from the pyroptotic BRAF-mutant melanoma cells induced by BRAF and MEK inhibitors via caspase-3/GSDME pathway, was proved to enhance the durative immunotherapeutic effects by inducing T-cell proliferation and infiltration, which might be a potential strategy against tolerance of both BRAF and MEK inhibitors in patients with BRAF-mutant melanoma (Erkes et al., 2020). Additionally, photodynamic treatment (PDT), an emerging treatment strategy, was also reported to enhance the infiltration of cytotoxic T lymphocytes (CTLs) by inducing pyroptosis, thus strengthening the anti-tumor effects (Lu et al., 2021). Therefore, stimulating anti-cancer immunity by mediating pyroptosis shows an excellent prospect in the treatment of malignancies. However, relevant studies are still insufficient in PC.

In this work, we established and validated a novel prognostic signature of PAAD that utilized 10 PRlncRNAs, including TRAF3IP2-AS1, LINC00519, LINC01133, LINC02251, AC005332.6, AL590787.1, AC090114.2, TRPC7-AS1, MIR223HG, and MIR3142HG. TRAF3IP2-AS1, known as an N6-methlydenosine-related lncRNA, performed anti-tumor effects in *NONO-TFE3* translocation renal cell carcinoma (*NONO-TFE3* tRCC) by reducing the stability of PARP1 [poly (ADP-ribose) polymerase 1] and elevating the expression level of PTEN (phosphatase and tensin homolog) (Yang et al., 2021). LINC00519 was overexpressed and related to a poor prognosis in lung squamous cell carcinoma (LUSC) patients, which promoted tumor progression via miR-450b-5p/miR-515-5p/YAP1 pathway (Ye et al., 2020). Similarly, it was also demonstrated to stimulate tumor development through miR-876-3p/MACC1 axis by serving as an emulative endogenous RNA in patients with tongue squamous cell carcinoma (TSCC) (Liu et al., 2020). And LINC01133, a widely reported carcinogenic lncRNA, was shown to exert promoting effects in multiple human malignancies, which was also associated with tumor proliferation and epithelial–mesenchymal transformation (EMT) in PC (Huang et al., 2018; Liu and Xi, 2020; Zhai et al., 2020; Liu et al., 2021; Yin et al., 2021; Sun et al., 2022). TRPC7-AS1 was up-regulated in hepatitis B virus-related HCC tissues, promising to serve as a novel biomarker for HCC (Zhu et al., 2021). Moreover, AC090114.2, MIR223HG, and MIR3142HG were also described as potential prognostic predictors in patients with cholangiocarcinoma (CCA), lung adenocarcinoma (LUAD), and head and neck squamous cell carcinoma (HNSCC), respectively (Chen et al., 2021; Geng et al., 2021; Xie et al., 2021). Therefore, 10 prognostic lncRNAs uncovered in our study may play a critical part in modulating the progression of PAAD as well, which may attribute to their functions in mediating pyroptosis. However, other than LINC01133, the rest of lncRNAs have not been reported in PAAD, thus further research remains needed.

In total, pyroptosis as an emerging type of PCDs has been shown to participate in regulating tumor development through various mechanisms, which displays inconstant effects in different cancers. From one perspective, pyroptosis suppresses tumor growth by stimulating cancer cell death, and from another perspective, it also contributes to tumor progression by inducing a tumor conducive microenvironment (Janowski et al., 2013; Tang et al., 2020). Combining pyroptosis inducers with immunotherapy displays a considerable prospect in certain resistant tumors, promising to be a valuable strategy for cancer treatment (Janowski et al., 2013; Tang et al., 2020). In this work, we constructed a novel PRlncRNAs-based risk model by integrating the genomic data aiming to assist the prognosis prediction of PAAD. Additionally, a significant correlation was observed among ICGs, immune cells, and our risk signature; the poor survival of patients with high-risk scores might be caused by the reduction in immune cells infiltration and immunocompetence, and those with low-risk scores possessed higher infiltration levels that might be prone to benefit from ICIs treatment, implying that our risk model might be associated with the TIME and is promising to assist in the treatment and management of PAAD. However, further experiments are required to verify the moderating effects of these PRlncRNAs in PAAD. Finally, there are still some deficiencies in this study. For example, our risk model has been validated internally in the TCGA database, whereas the other external validation was lacking, and validation in clinical samples is still needed in the future. Thus, further exploration is required to refine our prognostic signature.

## Conclusion

This work is the first to investigate the significance of PRlncRNAs in prognosis prediction and their intrinsic relations with the immune microenvironment of PAAD. The results indicate that high-risk PAAD patients suffer a poorer prognosis compared to those at low risk, and with regard to tumor immune microenvironment, low-risk PAAD patients have higher levels of immune cells infiltration that maybe more likely to benefit from immunotherapeutic, which is possibly attributed to the increase in pyroptotic cell death mediated by PRlncRNAs, implying that pyroptosis inducing may be a potential therapeutic to enhance the immunotherapy response in PAAD.

## Data Availability

The original contributions presented in the study are included in the article/Supplementary Material, and further inquiries can be directed to the corresponding authors.

## Glossary

AUC: Area under the curve
ATP: adenosine-triphosphate
CNV: copy number variation
CRC: colorectal cancer
CTLs: cytotoxic T lymphocytes
CCA: cholangiocarcinoma
DAMPs: damage-associated molecular patterns
EMT: epithelial–mesenchymal transformation
FPKM: fragments per kilobase of transcript per million fragments mapped data
GSVA: gene set variation analysis
GSEA: gene set enrichment analysis
GO: gene ontology
HMGB1: high mobility group protein 1
HCC: hepatocellular carcinoma
HNSCC: head and neck squamous cell carcinoma
ICGs: immune checkpoint genes
IC50: lower half inhibitory concentration
ICIs: immune checkpoint inhibitors
IL18: interleukin 18
IL1β: interleukin 1 beta
K-M: Kaplan–Meier
KEGG: Kyoto Encyclopedia of Genes and Genomes
Lasso: least absolute shrinkage and selection operator
LncRNA: long non-coding RNA
LUSC: lung squamous cell carcinoma
LUAD: lung adenocarcinoma
MEG3: maternally expressed gene 3
NEAT1: nuclear paraspeckle assembly transcript 1
NMF: non-negative matrix factorization
NKs: natural killer cells
PAAD: pancreatic adenocarcinoma
PAMPs: pathogen-associated molecular patterns
PDT: photodynamic treatment
PARP1: poly (ADP-ribose) polymerase 1
PTEN: phosphatase and tensin homolog
PC: pancreatic cancer
PCD: programmed cell death
PRlncRNAs: pyroptosis-related lncRNAs
PRGs: pyroptosis-related genes
ROC: receiver operating characteristic
ssGSEA: single-sample gene set enrichment analysis
TMB: tumor mutation burden
TIME: tumor immune microenvironment
TCGA: the Cancer Genome Atlas dataset
TPM: transcripts per kilobase million values
TSCC: tongue squamous cell carcinoma
