# Supplementary material for: A Comprehensive Analysis of Pyroptosis-Related lncRNAs Signature Associated With Prognosis and Tumor Immune Microenvironment of Pancreatic Adenocarcinoma
Source: Front Genet. 2022 Jul 6;13:899496. doi: 10.3389/fgene.2022.899496 (PMC9296806; doi:10.3389/fgene.2022.899496)
Supplement: Supplementary file 1 [file Table1.DOCX]

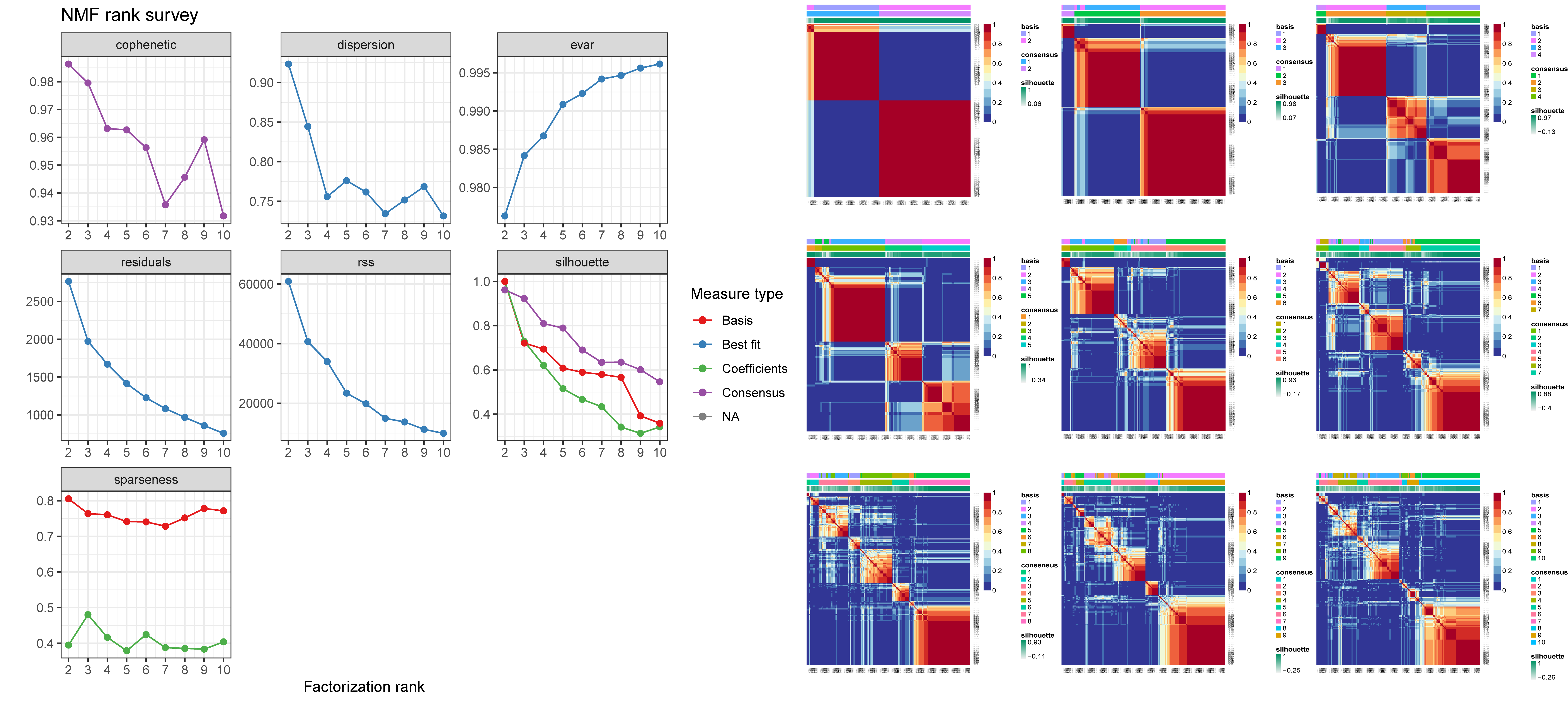


**Supplementary Figure 1**

Non-negative matrix factorization (NMF) clustering analysis of PAAD patients. The best clustering result was observed when rank=2 in 10 iterations, the degree of intergroup aggregation was minimal while the intragroup aggregation degree was significant under such condition.
