# Supplementary material for: A Comprehensive Analysis of Pyroptosis-Related lncRNAs Signature Associated With Prognosis and Tumor Immune Microenvironment of Pancreatic Adenocarcinoma
Source: Front Genet. 2022 Jul 6;13:899496. doi: 10.3389/fgene.2022.899496 (PMC9296806; doi:10.3389/fgene.2022.899496)
Supplement: Supplementary file 2 [file Table2.DOCX]

**Supplementary Figure 2**

Differential expression of pyroptosis-related genes between the patient subtypes classified by pyroptosis-related lncRNAs. As the result displayed that several pyroptosis-related genes showed a higher expression level in Cluster C1 compared to Cluster C2, including CHMP2A, CHMP4B, CHMP4C, GSDMB, CASP4, CASP5, CASP6, CASP8, IRF1, PYCARD, GPX4, CYCS, BAX, and BAK1. (*p < 0.05; **p < 0.01; ***p < 0.001)
