# Supplementary material for: A Comprehensive Analysis of Pyroptosis-Related lncRNAs Signature Associated With Prognosis and Tumor Immune Microenvironment of Pancreatic Adenocarcinoma
Source: Front Genet. 2022 Jul 6;13:899496. doi: 10.3389/fgene.2022.899496 (PMC9296806; doi:10.3389/fgene.2022.899496)
Supplement: Supplementary file 3 [file Table5.DOCX]

**Supplementary Table 1 52 pyroptosis-related genes**

| Gene | Type |
| --- | --- |
| \| BAK1 \| \| --- \| \| BAX \| \| CASP1 \| \| CASP3 \| \| CASP4 \| \| CASP5 \| \| CHMP2A \| \| CHMP2B \| \| CHMP3 \| \| CHMP4A \| \| CHMP4B \| \| CHMP4C \| \| CHMP6 \| \| CHMP7 \| \| CYCS \| \| ELANE \| \| GSDMD \| \| GSDME \| \| GZMB \| \| HMGB1 \| \| IL18 \| \| IL1A \| \| IL1B \| \| IRF1 \| \| IRF2 \| \| TP53 \| \| TP63 \| \| AIM2 \| \| CASP6 \| \| CASP8 \| \| CASP9 \| \| GPX4 \| \| GSDMA \| \| GSDMB \| \| GSDMC \| \| IL6 \| \| NLRC4 \| \| NLRP1 \| \| NLRP2 \| \| NLRP3 \| \| NLRP6 \| \| NLRP7 \| \| NOD1 \| \| NOD2 \| \| PJVK \| \| PLCG1 \| \| PRKACA \| \| PYCARD \| \| SCAF11 \| \| TIRAP \| \| TNF \| \| GZMA \| | \| pyroptosis \| \| --- \| \| pyroptosis \| \| pyroptosis \| \| pyroptosis \| \| pyroptosis \| \| pyroptosis \| \| pyroptosis \| \| pyroptosis \| \| pyroptosis \| \| pyroptosis \| \| pyroptosis \| \| pyroptosis \| \| pyroptosis \| \| pyroptosis \| \| pyroptosis \| \| pyroptosis \| \| pyroptosis \| \| pyroptosis \| \| pyroptosis \| \| pyroptosis \| \| pyroptosis \| \| pyroptosis \| \| pyroptosis \| \| pyroptosis \| \| pyroptosis \| \| pyroptosis \| \| pyroptosis \| \| pyroptosis \| \| pyroptosis \| \| pyroptosis \| \| pyroptosis \| \| pyroptosis \| \| pyroptosis \| \| pyroptosis \| \| pyroptosis \| \| pyroptosis \| \| pyroptosis \| \| pyroptosis \| \| pyroptosis \| \| pyroptosis \| \| pyroptosis \| \| pyroptosis \| \| pyroptosis \| \| pyroptosis \| \| pyroptosis \| \| pyroptosis \| \| pyroptosis \| \| pyroptosis \| \| pyroptosis \| \| pyroptosis \| \| pyroptosis \| \| pyroptosis \| |
