# Supplementary material for: A Comprehensive Analysis of Pyroptosis-Related lncRNAs Signature Associated With Prognosis and Tumor Immune Microenvironment of Pancreatic Adenocarcinoma
Source: Front Genet. 2022 Jul 6;13:899496. doi: 10.3389/fgene.2022.899496 (PMC9296806; doi:10.3389/fgene.2022.899496)
Supplement: Supplementary file 4 [file Table3.DOCX]

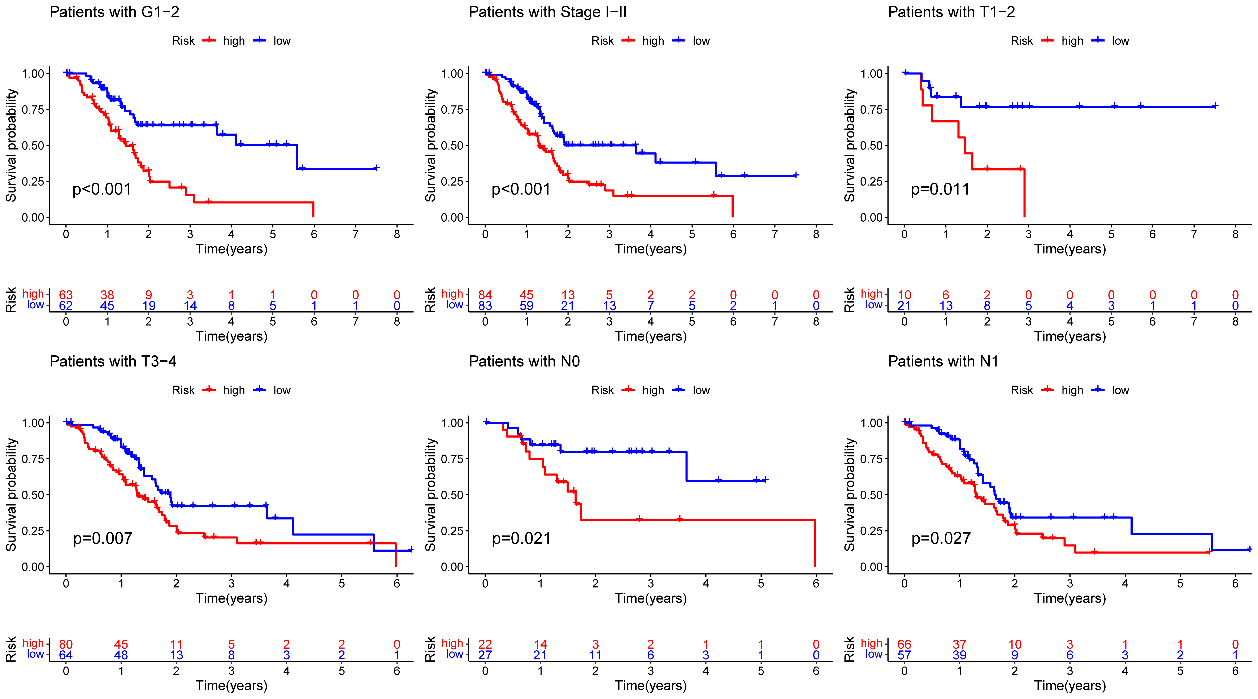


**Supplementary Figure 3**

K-M survival curves of patients with different clinical features between the high/low-risk groups. A significant survival discrepancy was observed in patients with tumor grade (G1-2), stage (Stage I-II), T stage (T1-2 and T3-4), as well as N stage (N0 and N1) between the two risk groups.
