# Supplementary material for: A Comprehensive Analysis of Pyroptosis-Related lncRNAs Signature Associated With Prognosis and Tumor Immune Microenvironment of Pancreatic Adenocarcinoma
Source: Front Genet. 2022 Jul 6;13:899496. doi: 10.3389/fgene.2022.899496 (PMC9296806; doi:10.3389/fgene.2022.899496)
Supplement: Supplementary file 5 [file Table4.DOCX]

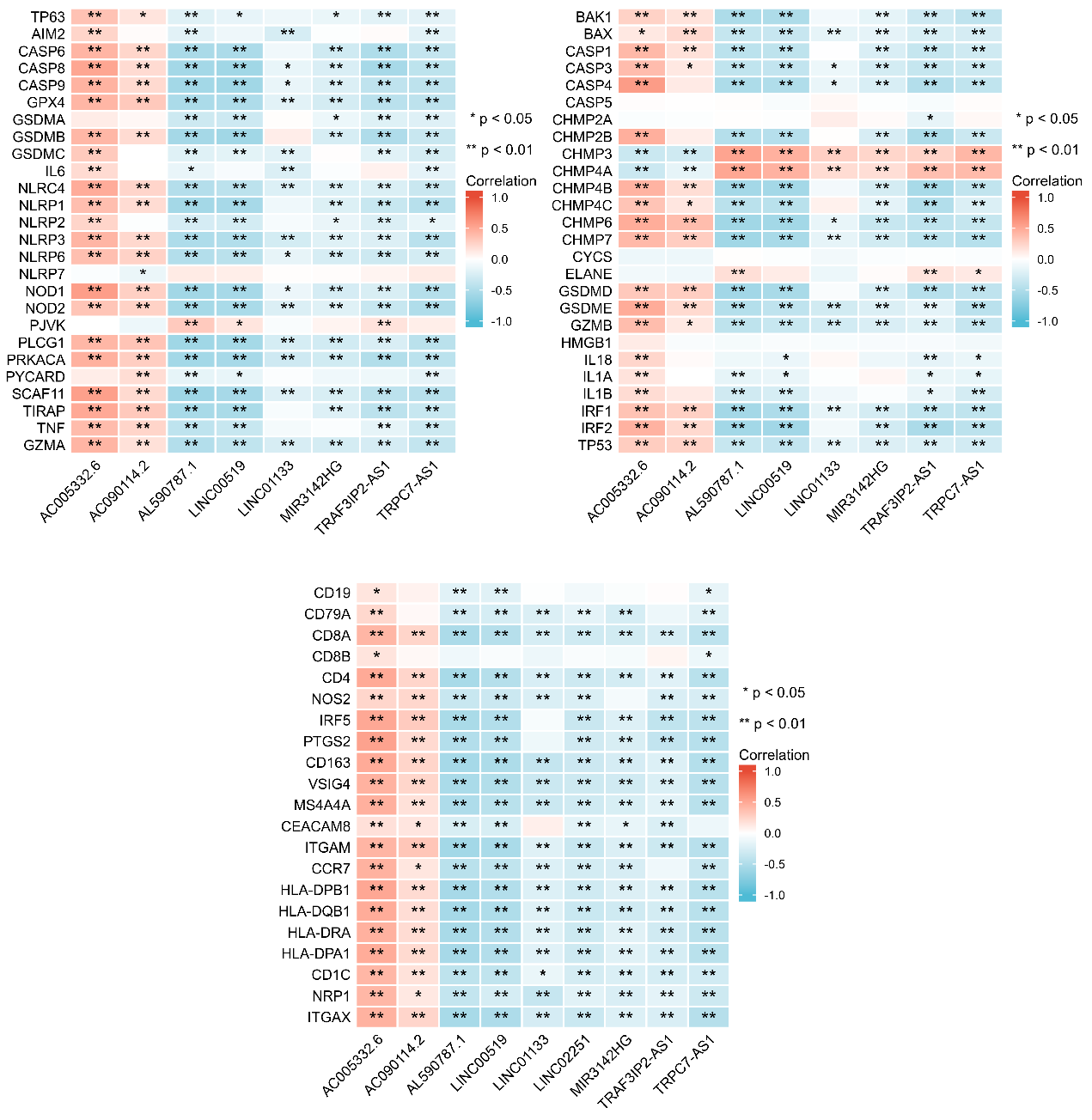


**Supplementary Figure 4**

Validation of the correlation between pyroptosis-related lncRNAs (PRlncRNAs) and pyroptosis-related genes (PRGs) as well as biomarkers of several immune cells. Correlation between PRlncRNAs and 52 PRGs (upper) and the relationship between PRlncRNAs and immunomarkers (below). To be specific, CD19 and CD79A represent B cells, CD8A and CD8B represent CD8+ T cells, CD4 represents CD4+ T cells, NOS2, IRF5, and PTGS2 represent M1 macrophages, CD163, VSIG4, and MS4A4A represent M2 macrophages, CEACAM8, ITGAM, and CCR7 represent Neutrophils, HLA-DPB1, HLA-DQB1, HLA-DRA, HLA-DPA1, CD1C, NRP1, and ITGAX represent Dendritic cells, respectively. The results validated that there was indeed a tight and complicated association between PRlncRNAs and these genes. (*p < 0.05; **p < 0.01)
