# Supplementary material for: A Comprehensive Analysis of Pyroptosis-Related lncRNAs Signature Associated With Prognosis and Tumor Immune Microenvironment of Pancreatic Adenocarcinoma
Source: Front Genet. 2022 Jul 6;13:899496. doi: 10.3389/fgene.2022.899496 (PMC9296806; doi:10.3389/fgene.2022.899496)
Supplement: Supplementary file 6 [file Table6.DOCX]

**Supplementary Table 2 Result of univariate cox analysis of PRlncRNAs.**

| Gene | HR | HR.95L | HR.95H | Pvalue |
| --- | --- | --- | --- | --- |
| AC013400.1  AC027575.2  AC005281.1  AC090241.3  AL672291.1  LINC02323  LRRC8C-DT  AC138356.1  AC009065.2  AC080013.4  TRIM52-AS1  RFX5-AS1  AC010175.1  AC009065.5  PDCD4-AS1  AC090948.3  AL390208.1  ST3GAL5-AS1  AC079385.3  PCAT7  AFAP1-AS1  TRAF3IP2-AS1  FLG-AS1  AC021242.3  LINC01410  FGF14-AS2  TMEM254-AS1  ZNF236-DT  AC002059.1  LINC00519  AC005089.1  AF111169.3  LINC01133  AC100810.1  LINC02251  AC005062.1  AC005332.6  LINC02593  LINC00857  AC087501.4  LINC02044  AL122010.1  AC096733.2  AL590787.1  LINC01091  MEG3  AC090114.2  TMEM105  AL158163.2  AP003559.1  AP000802.1  AP000894.4  AC005498.2  ZBED3-AS1  LINC00847  TRPC7-AS1  PAN3-AS1  AC022098.1  AC012213.4  LINC02600  LINC02041  MIR223HG  Z97832.2  AL355312.3  AL354809.1  MIR3142HG  AC078923.1  AP005233.2  HEIH  SUGT1P4-STRA6LP  AC016876.2  AC016590.1  AC009812.1  AC009159.3  ZNF582-AS1 | 0.303697885  0.824561659  0.688428564  0.058512485  0.310421581  1.108279586  0.53766646  0.234840876  1.011095579  0.442057389  0.953272631  0.803656788  0.750753237  1.011620523  0.899430445  0.824659093  0.599124293  0.540693105  0.06866322  1.080250934  1.011554982  0.544515494  0.592262351  0.221363019  0.749350882  0.906589562  0.630779291  0.628702031  0.730990726  1.160537265  0.405370759  0.581371112  1.005150094  0.959411114  0.410519576  0.578973152  0.971297971  0.886648518  1.0473218  0.401913991  0.047762518  0.846869996  0.608716935  0.142708487  0.624644041  0.979618811  0.803423149  1.101679913  0.529596155  0.779162066  0.024745288  0.900518971  0.418733347  0.314907559  0.965132932  0.827945714  0.698305376  0.624820302  0.870221881  0.76659113  1.015847238  0.934864117  0.353275389  1.004785803  0.250872868  0.889594103  1.376437818  1.003699035  0.982109253  0.330994112  0.577673253  0.274772426  0.814322523  0.800047166  0.816342325 | 0.125540499  0.731724039  0.480177788  0.00673318  0.11693746  1.027688588  0.356512934  0.075312107  1.001155398  0.230819396  0.926480869  0.666163271  0.584334408  1.002394252  0.839200187  0.6917295  0.446015722  0.33295382  0.010644495  1.000446127  1.001563026  0.392620201  0.373594776  0.081055811  0.593819983  0.850884223  0.467027611  0.500625239  0.597437382  1.027832422  0.188225828  0.42572534  1.002576888  0.927318745  0.185470771  0.389158703  0.946943416  0.793682458  1.012685048  0.247665271  0.006768177  0.773201832  0.466853369  0.029619852  0.456495231  0.963719814  0.707671807  1.007384221  0.367650173  0.639886548  0.001985543  0.840002654  0.212962079  0.139913366  0.941430494  0.72134399  0.582159566  0.462337223  0.784659042  0.613206438  1.002993598  0.877010392  0.215406612  1.000313213  0.09670765  0.794228447  1.091899521  1.000713353  0.971732566  0.16669946  0.341292025  0.083652273  0.722796071  0.678134643  0.709298093 | 0.734682481  0.92917807  0.986996691  0.508483463  0.824043536  1.195190504  0.810868821  0.732289129  1.021134453  0.846613148  0.98083915  0.969528433  0.964568259  1.020931714  0.96398349  0.983133754  0.804792074  0.878046794  0.442917928  1.166421709  1.021646622  0.755175416  0.938917554  0.604541309  0.945617798  0.965941795  0.851946448  0.789545178  0.894399075  1.310375811  0.873022865  0.793921193  1.007729905  0.992614126  0.908640866  0.861370716  0.996278903  0.990503931  1.083143228  0.65223055  0.337056498  0.927557024  0.793688836  0.687569682  0.854730022  0.995780102  0.912130099  1.204802105  0.762877616  0.948751817  0.308393844  0.965395066  0.823327875  0.708772677  0.989432128  0.950301264  0.837623268  0.844406182  0.965114886  0.958342776  1.028865601  0.996534278  0.579385652  1.00927839  0.650798521  0.996410632  1.735124002  1.006693626  0.992596749  0.657213299  0.977773764  0.902544348  0.917438815  0.943876668  0.939541214 | 0.008193016  0.001549527  0.042237517  0.010081232  0.018849668  0.007607435  0.003075861  0.012527013  0.028593797  0.013808684  0.001001503  0.022418332  0.024954711  0.01345361  0.002724681  0.031584213  0.000668269  0.012929556  0.004859806  0.048683334  0.023309375  0.000269719  0.025876645  0.003263067  0.015054125  0.002437749  0.002657431  6.52E-05  0.002333298  0.016258647  0.02106075  0.000645975  8.57E-05  0.016985112  0.028070491  0.007013429  0.024595165  0.03327101  0.007047729  0.000224271  0.002282457  0.000344223  0.000245648  0.015228562  0.003271603  0.013644219  0.000723634  0.033912142  0.000641577  0.013007819  0.004054208  0.003155232  0.011619285  0.005245011  0.00515173  0.007256321  0.000109274  0.00220908  0.00847882  0.019621129  0.01551867  0.038783185  3.75E-05  0.035945161  0.004466824  0.043164357  0.006849789  0.015135823  0.000865043  0.001581058  0.040985769  0.033258472  0.000734229  0.008175869  0.004661019 |
